# Individuals with persisting post‐concussion symptoms with physiological subtype demonstrate altered cardiovascular and autonomic responses to face cooling

**DOI:** 10.1113/EP092583

**Published:** 2025-07-01

**Authors:** Phillip J. Wallace, Steve Lidstone, Josh G. Nowlan, Johnathan Ljubanovich, Brandon J. McKinlay, Stephen A. Klassen, Stephen S. Cheung

**Affiliations:** ^1^ Department of Kinesiology Brock University St Catharines Ontario Canada; ^2^ Brock University Sports Performance & Sports Medicine Brock University St Catharines Ontario Canada; ^3^ Faculty of Applied Health & Community Sciences Sheridan College Brampton Ontario Canada

**Keywords:** autonomic nervous system function, exercise intolerance, face cold pressor test, persisting post‐concussion symptoms, persisting symptoms after concussion

## Abstract

Individuals with persisting post‐concussion symptoms with physiological subtype (PPCS‐P) demonstrate exercise intolerance due to exacerbation of concussion‐like symptoms during incremental exercise. We tested the hypothesis that individuals with PPCS‐P (*n *= 12) would have a blunted cardiac autonomic response to face cooling compared to healthy controls (CTRL, *n *= 12). Participants were supine and performed a 5 min baseline, then experienced a 3 min face cold pressor test followed by 5 min of recovery. A three‐lead electrocardiogram was used to measure heart rate and root mean square of successive differences in R‐R intervals (RMSSD), finger photoplethysmography was used to measure mean arterial pressure (MAP), and laser‐Doppler flowmetry was used to measure finger skin blood flux. The PPCS‐P group had a lower exercise tolerance (9.9 ± 3.2 min, *P *< 0.001) and lower peak heart rate (170.0 ± 14.0 beats·min^−1^, *P *< 0.001) compared to CTRL (19.6 ± 2.5 min; 193.0 ± 5.0 beats·min^−1^). PPCS‐P demonstrated a blunted mean heart rate (CTRL: ∆−4.0 ± 5.0 beats·min^−1^, PPCS‐P: ∆2.0 ± 4.0 beats·min^−1^; group effect: *P *< 0.001) and mean RMSSD (CTRL: ∆26.6 ± 34.7 ms, PPCS‐P: ∆−1.8 ± 33.9 ms; group effect: *P* = 0.026) responses at 2 min of face cooling compared to CTRL. Both groups had a significant increase in MAP during face cooling, where at 2 min, MAP was higher in PPCS‐P (∆+13.2 ± 5.5 mmHg) compared to CTRL (∆+8.7 ± 6.9 mmHg, group effect: *P *< 0.001). Furthermore, PPCS‐P had a sustained lower finger skin blood flux (group effect: *P *< 0.001) during face cooling (PPCS‐P: ∆−48.2 ± 27.1%, CTRL: ∆−12.8 ± 24.7% at 2 min). These data suggest that individuals with PPCS‐P demonstrate altered cardiac and peripheral autonomic function during face cooling compared to healthy controls.

## Abstract (highlights)

- **What is the central question of this study?**
  Are there physiological differences in responses to face cooling between individuals who are apparently healthy versus those with persisting post‐concussion symptoms with physiological subtype?
- **What is the main finding and its importance?**
  Compared to controls, individuals with persisting post‐concussion symptoms with physiological subtype exhibited altered autonomic and cardiovascular responses to a face cold pressor test including blunted heart rate variability responses, greater blood pressure responses, and greater finger skin blood flux reductions to face cooling. Persisting post‐concussion symptoms with physiological subtype do not appear to affect cerebral autoregulation. These data suggest that impaired autonomic regulation of the heart and vasculature may mechanistically contribute to persisting post‐concussion symptoms in humans.

## 1 INTRODUCTION

Sports‐related concussions are traumatic brain injuries that have transient cognitive, emotional and physiological effects (McCrory et al., 2017; Patricios et al., 2023). While a majority of individuals will recover in the initial 10–14 days following a sports‐related concussion, ∼10–30% of individuals will demonstrate persisting symptoms after a concussion greater than 4 weeks following injury (formally known as post‐concussion syndrome or post‐concussion disorder/dysfunction) (Broshek et al., 2022; Ellis et al., 2015; Patricios et al., 2023; Yeates et al., 2023). With persisting symptoms after a concussion, there are different subtypes including: (1) physiological, (2) cervogenic, and (3) vestibular‐occular (Ellis et al., 2016, 2015). The focus of this study will be the persisting post‐concussion symptoms with physiological subtype (PPCS‐P). Individuals with PPCS‐P are demonstrated to be asymptomatic or have low symptoms at rest, but individualized concussion‐like symptoms (e.g. nausea, headache, pressure) emerge and increase in severity during exercise proportional to exercise intensity (Clausen et al., 2016; Ellis et al., 2015; Leddy et al., 2011, 2010, 2021; Mercier et al., 2024; Willer & Leddy, 2006). A key feature is that individuals with PPCS‐P demonstrate exercise intolerance with reduced exercise capacity due to concussion‐like symptoms (Leddy et al., 2010).

The underlying pathophysiology remains unclear; however, PPCS‐P is hypothesized to be caused by altered autonomic nervous system function and the control of cerebral blood flow (demonstrated during cerebrovascular reactivity test and incremental exercise) (Clausen et al., 2016; Ellis et al., 2015; Leddy et al., 2017, 2018). Paradoxically, although concussion‐like symptoms are exacerbated with exercise, aerobic exercise has emerged as a therapeutic rehabilitation strategy to facilitate recovery (Baker et al., 2012; Clausen et al., 2016; Haider et al., 2019; Leddy & Willer, 2013; Leddy et al., 2013, 2010; Mercier et al., 2024). A typical PPCS‐P prescription commonly includes aerobic exercise for 20 min, five to seven times per week at an intensity that can range between 70% and 90% (70–80% most common) of the symptomatic heart rate threshold (Ellis et al., 2015; Leddy et al., 2017, 2018). Sub‐symptom threshold aerobic exercise allows for an individualized approach to PPCS‐P rehabilitation to account for inter‐individual variability in PPCS‐P symptoms and fitness levels (Ellis et al., 2015). Six to twelve weeks of sub‐symptom aerobic exercise training has been demonstrated to improve/restore exercise tolerance, minimize PPCS‐P symptoms at rest and during exercise (Baker et al., 2012; Clausen et al., 2016; Kozlowski et al., 2013; Leddy et al., 2018, 2010; Mercier et al., 2024), restore mean cerebral blood velocity in the middle cerebral artery (MCA_v,mean_) during exercise to control levels (Clausen et al., 2016) and improve quality of life measures (Mercier et al., 2024). Aerobic exercise is hypothesized to restore autonomic nervous system function in PPCS‐P through gradually increasing parasympathetic activity and decreasing sympathetic activity over time (Carter et al., 2003; Ellis et al., 2015; Leddy et al., 2018). Recently, it has been demonstrated that sub‐symptom aerobic exercise does not affect resting heart rate variability or blood pressure in PPCS‐P (Mercier et al., 2024). However, it may be necessary to stimulate the autonomic nervous system to reveal underlying differences in PPCS‐P. This limits our understanding of the alteration of the autonomic nervous system in PPCS‐P as well as the potential mechanisms for why sub‐symptom aerobic exercise works to rehabilitate PPCS‐P.

The face cold pressor test (FCPT) assesses both branches of the autonomic nervous system by placing a cold stimulus on the cheeks and/or forehead for 1–3 min. Cold stimulates the trigeminal nerve and transiently increases parasympathetic activity (∼3 min), leading to decreases in heart rate (Al‐Ani et al., 1995; Fisher et al., 2015; Gagnon et al., 2013; Heath & Downey, 1990; Hilz et al., 1999; Johnson et al., 2017; Schlader et al., 2016, 2018; Stemper et al., 2002) and increases in vagal‐mediated heart rate variability measures such as the square root of the mean successive differences in R‐R intervals (RMSSD) (Johnson et al., 2018, 2017; Schlader et al., 2016, 2018). Additionally, cold stress elicits a sustained increase in sympathetic activity, causing systemic peripheral vasoconstriction and increases in blood pressure (Al‐Ani et al., 1995; Fisher et al., 2015; Gagnon et al., 2013; Heath & Downey, 1990; Johnson et al., 2017; Schlader et al., 2016). Finger skin blood flow decreases (i.e. vasoconstricts) with face cooling, and gradually returns to baseline levels by 3 min of the FCPT and also returns to baseline during recovery when the cold stimulus is removed (Gagnon et al., 2013). During the FCPT, cerebral autoregulation appears to remain intact with small increases in MCA_v,mean_ and cerebrovascular resistance that is less than total peripheral resistance (Brown et al., 2003). In acutely concussed (non‐PPCS‐P) athletes (< 10 days post injury), there is an impaired FCPT response to both branches of the autonomic nervous system, where there is no change in heart rate or cardiac parasympathetic activity (i.e. RMSSD) and a blunted blood pressure response compared to healthy controls (Johnson et al., 2018). Blood pressure is also blunted during strong sympathetic‐mediated stimuli such as placing a hand in cold water, indicating that there is sympathetic dysfunction in recently concussed athletes (Johnson et al., 2020). It is currently unknown what the autonomic responses are to the FCPT in PPCS‐P.

The primary purpose of this study was to assess the cardiac autonomic nervous system and blood pressure responses in individuals who sustained a sport‐related concussion and demonstrated PPCS‐P compared to a control population during FCPT. A secondary purpose was to assess if there were differences in cerebral blood velocity or vasoconstriction during the FCPT. We hypothesize that similar to athletes who are acutely concussed (Johnson et al., 2020), individuals with PPCS‐P will have attenuated increases in both cardiac autonomic nervous system activation and blood pressure during face cooling compared to apparently healthy controls.

## 2 METHODS

### 2.1 Ethical approval

The experimental protocol and procedures were approved by the Bioscience Research Ethics Board at Brock University (REB no. 17‐385) and conformed to the latest revision of the *Declaration of Helsinki* (except clause 35 as the study was not registered). Prior to participating in the study, the study procedures, benefits and risks were explained to participants. Furthermore, participants provided both verbal and written consent. Participants were allowed to voluntarily terminate participation at any point in the study until data collection was complete, and all data were de‐identified. Participants provided ongoing verbal consent throughout data collection procedures.

### 2.2 Participants

Twelve (6 male, 6 females) apparently healthy controls (CTRL) and 12 (5 males, 7 females) individuals with PPCS‐P participated in this study (characteristics in Table 1). All participants were screened with a Physical Activity Readiness Plus Questionnaire and were self‐reported to be free from any cardiovascular, metabolic, neurological (other than concussion in PPCS‐P group), respiratory or endocrine disease. For CTRL, individuals were included if they had not sustained a concussion (diagnosed or suspected) in the previous 12 months and were recreationally active (≥150 min per week of moderate intensity exercise) or involved in competitive sport. Individuals in both groups were excluded from participating if they required a balance or gait aid or were taking any medications that could influence the autonomic nervous system (e.g. beta‐blockers, stimulants, antihypertensives, steroids). We had an age range inclusion criterion of 18–50 years of age for both groups, but all participants were drawn from the university community and the age range was 18–25 years. For the PPCS‐P group, individuals were referred into the study by the Brock University Sports Medicine Clinic. For the PPCS‐P group, inclusion criteria for this study were: (1) diagnosed with a sports‐related concussion by a physician in the previous 12 months, (2) had persisting post‐concussion symptoms diagnosed by physician and lasting greater than 2 weeks since injury, (3) received medical clearance to perform exercise in the study, and (4) demonstrated exercise intolerance during testing in the preliminary session. An additional exclusion criterion for the PPCS‐P group was the if the mechanism of injury was not a sports‐related concussion (e.g. motor vehicular accident, slip/fall). Therefore, the mechanism of injury for all individuals in PPCS‐P group occurred during participation in sport and consisted of impact to the head or upper body. The time from initial sports‐related concussion injury is provided in Table 1. The range of time from injury in this study was 3–48 weeks, 11/12 participants were 3–12 weeks post‐injury, and one participant was 48 weeks post‐injury. Note the timeline definition for persisting post‐concussion symptoms has been updated between consensus statements from the start of this study (>10–14 days in adults) (McCrory et al., 2017) to ≥4 weeks (Patricios et al., 2023) (although participants performed their first BCTT/BCBT 3 weeks post‐injury, they continued to be symptomatic past 4 weeks as participants participated in a minimum of 1 week of sub‐symptom aerobic exercise training with an improvement on the BCTT/BCBT (data not included). We elected to not match CTRL participants to the PPCS‐P group for specific sport participation to avoid the potential influence of repetitive non‐concussive head impacts on autonomic function (Mainwaring et al., 2018). We also did not match participants to individuals who had sustained a sport‐related concussion and recovered, or individuals who sustained a non‐related head injury (e.g. anterior cruciate ligament tear) (Yeates et al., 2023), as we were interested in first quantifying differences compared to healthy controls.

**TABLE 1:** Participant characteristics and exercise testing results.

	PPCS‐P	CTRL	*P*
Sex (male/female)	5/7	6/6	—
Age (years)	20.0 ± 2.0	21.0 ± 2.0	0.332
Height (cm)	176.0 ± 12.1	175.3 ± 11.7	0.887
Mass (kg)	77.4 ± 12.0	77.2 ± 11.9	0.972
Weeks since injury	11.0 ± 12.0	N/A	N/A
BESS (no. of errors)	16.0 ± 5.0	12.0 ± 4.0	0.021*
BCTT/BCBT stage completed (stage number)	9.5 (6.5–12.5	20 (17–22)	< 0.001*
Peak heart rate on BCTT/BCBT (beats·min^−1^)	170.0 ± 14.0	193 ± 5.0	< 0.001*

Data are presented as means ± SD and compared using Student's unpaired *t*‐test. BCTT/BCBT stage completed was compared using a Mann–Whitney test and is presented a median (quartile 1 – quartile 3). * indicates significant difference. BCBT, Buffalo Concussion Bicycle Test; BCTT, Buffalo Concussion Treadmill Test; BESS, Balance Errors Scoring System.

### 2.3 Experimental design

The study consisted of a preliminary assessment and an experimental assessment. The preliminary assessment consisted of collecting anthropometric data, balance testing, and performing the Buffalo Concussion Treadmill Test (BCTT) or a modified Buffalo Concussion Bike Test (BCBT). For the PPCS‐P group, the preliminary session was used to determine if individuals with persisting symptoms after a concussion demonstrated exercise intolerance to determine the physiological subtype. If they did not demonstrate exercise intolerance, they were excluded. Participants returned to the lab 2–5 days following the preliminary assessment and performed the FCPT protocol. Participants were asked to avoid exercise and alcohol 24 h and caffeine 12 h prior to each assessment. Each session was identical for both groups.

### 2.4 Preliminary assessment

Upon arrival, body mass (kg; GFK 330AH, Oxford, CT, Adam Equipment, USA) and height (cm; STAT7 7X, Ellard Instrumentation Ltd, Monroe, WA, USA) were recorded. Participants performed all testing with athletic clothes. They performed balance testing using the Balance Error Scoring System (BESS), which consisted of six separate 20 s balance tasks consisting of three stances (double‐leg, single leg, tandem) on two different surfaces (stable, unstable; Balance‐Pad, Airex AG, Sins, Switzerland) (Iverson et al., 2008). Participants had their hands on their hips and eyes closed throughout a test. The number of errors was recorded, with a higher number indicating worse balance performance (Iverson et al., 2008). This is a test that is included in the most recent Sport Concussion Assessment Tool 6 (SCAT6) (Echemendia et al., 2023).

A BCTT/BCBT test was performed on individuals with persisting post‐concussion symptoms to diagnose PPCS‐P by a registered kinesiologist for inclusion into the study. Prior to testing, individuals with persisting post‐concussion symptoms filled out a baseline (PRE‐BCTT/BCBT) measure of symptoms using the Post‐Concussion Symptom Scale assessment which is a 22‐question Likert scale (none = 0, moderate = 3–4, severe = 5–6) (Lovell et al., 2006). The symptoms covered in the questionnaire included physical (i.e. headache, nausea, fatigue, photophobia, etc.), cognitive (i.e. difficulty concentrating, mental fog), sleep disturbances, and emotional (i.e. sadness, feeling more emotional). The sum was recorded to index total symptom score and could range from 0 to 132. Next, participants were instrumented with a three‐lead electrocardiogram (MLA2340, ADInstruments, Colorado Springs, CO, USA) to measure heart rate. Participants then performed 5 min of seated rest followed by the BCTT/BCBT where choice of performing the test was based on participant comfort. The BCTT (*n *= 6 in PPCS‐P, *n *= 12 in CTRL) was performed on a treadmill where participants started walking at 5.3 km·h^−1^ (5.8 km·h^−1^ if taller than 178 cm) with a 0% incline (Clausen et al., 2016; Haider et al., 2019; Leddy & Willer, 2013; Leddy et al., 2010). Each minute the incline was increased by 1% until the incline reached 15% (at the 15 min mark), then the speed was increased by 0.6 km·h^−1^ each minute (Clausen et al., 2016; Haider et al., 2019; Leddy & Willer, 2013; Leddy et al., 2010). Alternatively, a modified BCBT (*n *= 6 in PPCS‐P, *n *= 0 in CTRL) was performed on a cycle ergometer (Velotron, RacerMate Inc., Seattle, WA, USA), where initial workload was calculated based on calculated gross oxygen consumption (Haider et al., 2019), then every 2 min the workload was increased by 15 W. Participants were allowed to freely choose their cadence, and a test termination criterion was the inability to maintain cadence above 60 rev. m^−1^ for 5 consecutive seconds. In CTRL, the testing was performed to volitional fatigue or if individuals reached their age‐predicted max heart rate (220 − age) (Leddy et al., 2017, 2018). For individuals with persisting post‐concussion symptoms, concussion‐related symptoms were measured at baseline and every 2 min of the BCTT/BCBT using a 0–10 visual analogue scale (VAS) for symptom severity or worsening of symptoms (Leddy & Willer, 2013). The test was performed until (1) volitional fatigue, (2) attaining age‐predicted max heart rate, or (3) demonstrated symptom‐limited exercise intolerance (defined as a ≥3‐point change in baseline VAS score) (Clausen et al., 2016; Haider et al., 2019; Leddy & Willer, 2013; Leddy et al., 2011, 2010, 2017, 2013, 2018). If individuals demonstrated exercise intolerance, they were determined to have PPCS‐P and were included in the study. If individuals could exercise to voluntary fatigue or age‐predicted max heart rate, they were excluded from the study as they did not meet the criteria of PPCS‐P. Following the BCTT/BCBT, all participants performed a 5 min warm down and then 25 min of supine rest with eyes under a blindfold. Participants in PPCS‐P filled out the post‐concussion symptom scale following warm down (post‐BCTT/BCBT) and following supine recovery. To compare BCTT and BCBT times, stage completed (stage number) was compared to account for different lengths of tests.

### 2.5 Experimental trial

Upon arrival, participants were instrumented (see below), and then rested quietly in a supine position for 10 min. Participants then performed a 5 min baseline, immediately followed by the 3 min FCPT, and a 5 min recovery period with eyes closed, dim lighting, and the instruction to breathe normally throughout. For the FCPT, a pliable plastic bag (Ziploc Freezer Bag, SC Johnson, Racine, WI, USA) filled with crushed ice and water that was placed bilaterally on the cheeks and nose. The bags were removed for the recovery period. Symptoms during the test were measured using the VAS recorded at Baseline and 3 min of the FCPT, and Post‐Concussion Syndrome Symptom Scores were recorded pre‐Baseline, and post‐test.

### 2.6 Instrumentation and data analysis

Data were continuously sampled and stored for offline analysis using Labchart (Version 8.1.25, ADInstruments, Colorado Springs, CO, USA). Data were analysed as peak or nadir values during the FCPT as well as in 60 s increments at minutes 3–4 of baseline, each minute of the FCPT (e.g. minute 1, 2, etc.), 1–2 min of recovery (R1), and minutes 4–5 of recovery (R5). Baseline was taken at minutes 3–4 to avoid an anticipatory response to starting the FCPT (e.g. preparing ice, counting down to start). The recovery period was used to see if there were differences in ANS stimulation recovery (as individuals with PPCS‐P have prolonged persisting post‐concussion symptoms following the cessation of exercise). The 1–2 min time point of the recovery was used to avoid potential confounding influences of removing the ice and checking in with participants. Face temperature (°C) was measured on the left cheek using a flexible T‐type thermocouple sampled at 4 Hz. Heart rate (beats·min^−1^) was measured with a three‐lead electrocardiogram sampled at 1 kHz. All R waves from the electrocardiogram were visually inspected for ectopic beats (with no ectopic beats present in the data set). Heart rate variability was measured as the root mean square of successive differences in R‐R intervals (RMSSD) and the percentage of times successive heartbeat intervals exceeded 50 ms (pNN50) as these are indirect measures of cardiac parasympathetic activity (Labchart 8.1.25 Heart Rate Variability Module, ADInstruments, Colorado Springs, CO, USA). Beat‐by‐beat blood pressure was sampled at 400 Hz from the left middle finger maintained at heart level using finger photoplethysmography (Nexfin, bmeye, Irvine, CA, USA) to determine MAP. Stroke volume (mL), cardiac output (calculated as heart rate × stroke volume, L·min^−1^), and total peripheral resistance (calculated as the quotient of MAP and cardiac output, mmHg·L·min^−1^) were derived via the blood pressure waveform (Bogert & Van Lieshout, 2005).

A 2 MHz pulsed transcranial Doppler ultrasound system (Doppler‐Box, Compumedics GmbH, Singen, Germany) sampled at 400 Hz was used to measure MCA_v,mean_ (cm·s^−1^). The probes were positioned over the temporal window and were held in place using a secure and comfortable head frame (M600 Headframe, Spencer Technologies, Seattle, WA, USA) (Willie et al., 2011). We also calculated cerebrovascular conductance (MCA_v,mean,conductance_) as the quotient of MCA_v,mean_ and MAP (cm·min^−1^·mmHg).

Laser Doppler flowmetry (PeriFlux 5010 laser‐Doppler perfusion monitor, PeriMed, Järfälla, Sweden)—with a right‐angle probe (Probe 457) sampled at 40 Hz was taped to the left second digit finger pad and the ventral forearm to measure flux as an index of skin blood flow (arbitrary perfusion units (PU)) and peripheral vasoconstriction. Data was expressed as percentage change from Baseline PU values as well as cutaneous vascular conductance (CVC, PU mmHg) where PU data were normalized to MAP and expressed as a percentage change from Baseline values. The fingerpad was used as it is covered in glabrous skin, where vasomotor tone is controlled by the sympathetic nervous system and the reduction in blood flow during cold stress is mediated by the activation of the adrenergic vasoconstrictor system (Hodges & Johnson, 2009; Hodges et al., 2019). The ventral forearm is covered in non‐glabrous skin, which is controlled by the adrenergic vasoconstrictor system, nitric oxide, various co‐transmitters, and α_2c_‐adrenergic receptor expression (Hodges & Johnson, 2009; Hodges et al., 2019).

### 2.7 Statistical analyses

For the preliminary assessment data, data were compared using Student's two‐tailed independent *t*‐test between CTRL and PPCS‐P (GraphPad Prism; GraphPad Software Inc., La Jolla, CA, USA). Post‐concussion symptom scores are presented as median (quartile 1 – quartile 3) and were assessed using a 1 × 3 (pre‐BCTT/BCBT vs. 5 min post‐BCTT/BCBT vs. 30 min recovery) Friedman's ANOVA with a *post hoc* comparison using Dunn's multiple comparison test.

All physiological data from the FCPT are presented as the mean ± SD. Nadir and peak data during the FCPT were compared using two‐tailed independent *t*‐tests (GraphPad Prism) with significance set at *P* < 0.05. Pre‐ and post‐FCPT symptoms were compared using Wilcoxon's matched paired signed rank test for the PPCS‐P group only. We compared absolute cardiovascular data at baseline using a two‐tailed independent *t*‐test. As there were no statistically significant differences in baseline measures (Table 2), delta (∆) changes from baseline were used for all physiological variables (except face temperature, pNN50 and skin blood flux data) to determine changes over time. Data were analysed using a 2 Group (CTRL vs. PPCS‐P) × Time (1 min vs. 2 min vs. 3 min vs. R1 vs. R5) linear mixed model using the *lme4* (Bates et al., 2014) function with a random effect for participant using R (version 4.2.2) using the RStudio environment (Version 2023.03.1.446). If significant, a Bonferroni *post hoc* analysis was used to test for specific main effects between groups and time using the *emmeans* function (in the *emmeans* package) (Lenth, 2022). Data were assessed for normality through visual inspection of Q‐Q plots and homoscedasticity through visual inspection of homoscedasticity plots.

**TABLE 2:** Baseline absolute cardiovascular values compared between groups using unpaired *t*‐tests.

	PPCS‐P	CTRL	*P*
Heart rate (beats·min^−1^)	66.0 ± 7.0	65.0 ± 10.0	0.674
RMSSD (ms)	63.6 ± 31.1	93.9 ± 56.5	0.118
pNN50 (%)	38.2 ± 21.0	49.3 ± 24.8	0.251
Mean arterial pressure (mmHg)	87.2 ± 5.7	85.5 ± 8.1	0.606
Systolic blood pressure (mmHg)	118.0 ± 12.0	117.0 ± 10.0	0.877
Diastolic blood pressure (mmHg)	69.0 ± 6.0	68.0 ± 6.0	0.614
Total peripheral resistance (mmHg·L·min^−1^)	12.9 ± 1.9	13.2 ± 1.7	0.717
Stroke volume (mL)	104.9 ± 20.8	111.9 ± 16.4	0.437
Cardiac output (L·min^−1^)	6.8 ± 1.1	6.8 ± 1.5	0.994
Cerebral blood velocity in the middle cerebral artery (cm·s^−1^)	68.1 ± 10.3	72.3 ± 14.1	0.471

Data are presented as means ± SD. pNN50, percentage of number of times successive heart peat intervals exceed 50 ms; RMSSD, root mean square of successive differences in R‐R interval.

## 3 RESULTS

### 3.1 Preliminary assessment

A total of 26 participants with persisting post‐concussion symptoms were referred to the study to perform the BCTT/BCBT. A total of 12 participants were determined to have PPCS‐P with a significantly lower exercise tolerance, lower maximal heart rate achieved during the BCTT/BCBT, and secondary signs such as worse balance scores compared to CTRL (Table 1). The other 14 of these participants did not demonstrate exercise intolerance (median stage completed: 19.0 (17.0–20.0) min, no change in VAS) and were excluded from the study. In PPCS‐P, post‐concussion symptom scores (*P* = 0.002) increased from pre‐BCTT/BCBT (14 (7.5–34)) to post‐BCTT/BCBT (21 (16–36), *P* = 0.009) and returned to pre‐BCTT/BCCT levels following 30 min recovery (13 (11–20), *P* = 1.00).

### 3.2 Nadir and peak cardiovascular responses

**FIGURE 1:**
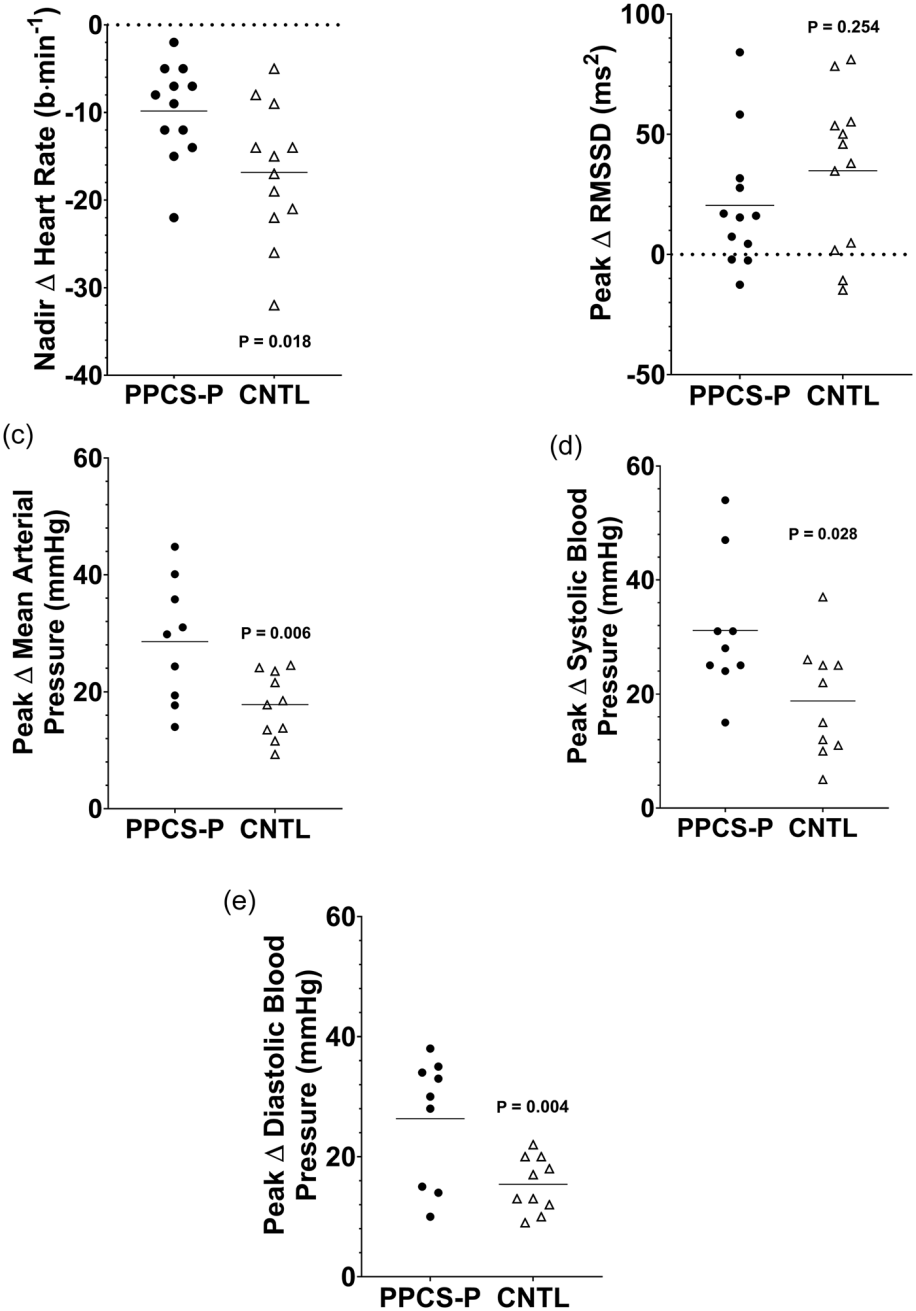
Delta (**∆**) individual and mean (horizontal bar) values for nadir heart rate (a), peak root mean square of successive differences in R‐R intervals (RMSSD) (b), peak mean arterial pressure (c), peak systolic blood pressure (d), and diastolic blood pressure (e) between individuals with physiological post‐concussion syndrome (PPCS‐P) and healthy controls (CTRL) during the 3 min face cold pressor test.

There was a greater FCPT response for ∆ heart rate nadir (Figure 1a) in CTRL (−17.0 ± 8.0 beats·min^−1^) compared to PPCS‐P (−10.0 ± 5.0 beats·min^−1^). There were no differences in ∆ peak RMSSD (Figure 1b, CTRL: (20.4 ± 27.1 ms, PPCS‐P: (34.8 ± 32.6 ms). Overall, there were greater ∆ peak blood pressure responses (Figure 1c–e) in PPCS‐P (∆ peak MAP: 28.5 ± 10.5 mmHg, ∆ peak systolic blood pressure (SBP): 31.0 ± 12.0 mmHg, ∆ peak diastolic blood pressure (DBP): 26.0 ± 11.0 mmHg) compared to CTRL (∆ MAP: 17.8 ± 5.5 mmHg, ∆ SBP: 19.0 ± 10.0 mmHg, ∆ DBP: 15.0 ± 5.0 mmHg) (all *P* ≤ 0.031).

### 3.3 Thermal responses

**FIGURE 2:**
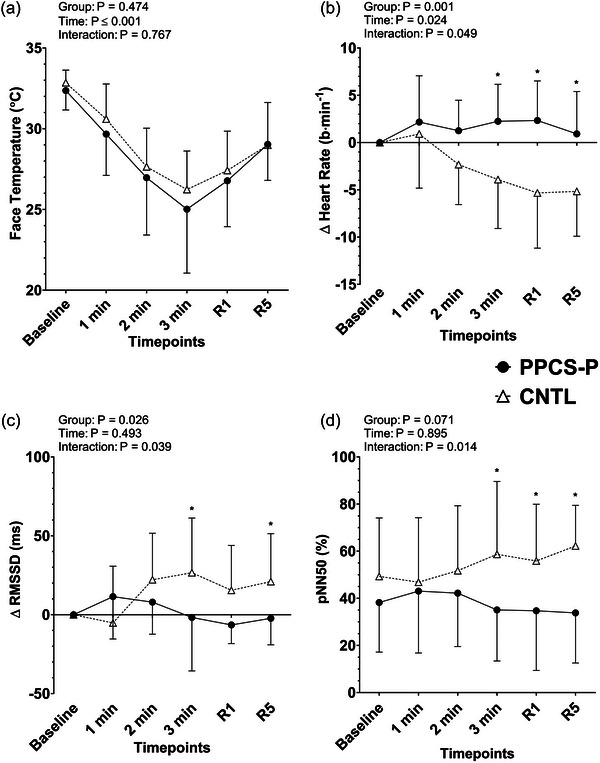
Statistical outputs from 2 (Group) × 5 (Time) linear mixed model, means and standard deviation for face temperature (a), heart rate (b), root mean square of successive differences in R‐R intervals (RMSSD) (c), and percentage of times successive heartbeat intervals exceed 50 ms (pNN50) (d). * indicates significant difference at specific timepoint.

Face temperature (Figure 2a) was not different between groups, nor was there an interaction effect. There was a time effect where face temperature decreased from Baseline (CTRL: 32.9 ± 0.8°C, PPCS‐P: 32.4 ± 1.2°C) to 3 min of the FCPT (CTRL: 26.2 ± 2.4°C, PPCS‐P: 25.0 ± 4.0°C at 3 min, *P* ≤ 0.001), and increased during recovery (CTRL: 29.0 ± 2.6°C, PPCS‐P: 29.0 ± 2.3°C, *P* ≤ 0.001 at R5 compared to 3 min); however, it was still less than Baseline temperature (*P* ≤ 0.001).

### 3.4 Cardiovascular responses

There were no differences between PPCS‐P and CTRL for baseline cardiovascular variables (Table 2). Heart rate (Figure 2b) demonstrated a group, time and interaction effect, where heart rate was lower in CTRL at 3 min (*P* = 0.002), R1 (*P* ≤ 0.001) and R5 (*P* = 0.002) compared to PPCS‐P. Concurrently, RMSSD (Figure 2c) demonstrated a group and interaction effect, where RMSSD was higher in CTRL compared to PPCS‐P at 3 min of the FCPT (*P* = 0.014) and R5 (*P* = 0.043), but not at R1 (*P* = 0.056). There was an interaction (*P* = 0.014) effect for pNN50 (Figure 2d), where pairwise comparisons revealed higher pNN50 in CTRL at 3 min (*P* = 0.024), R1 (*P* = 0.042) and R5 (*P* = 0.008) compared to PPCS‐P.

**FIGURE 3:**
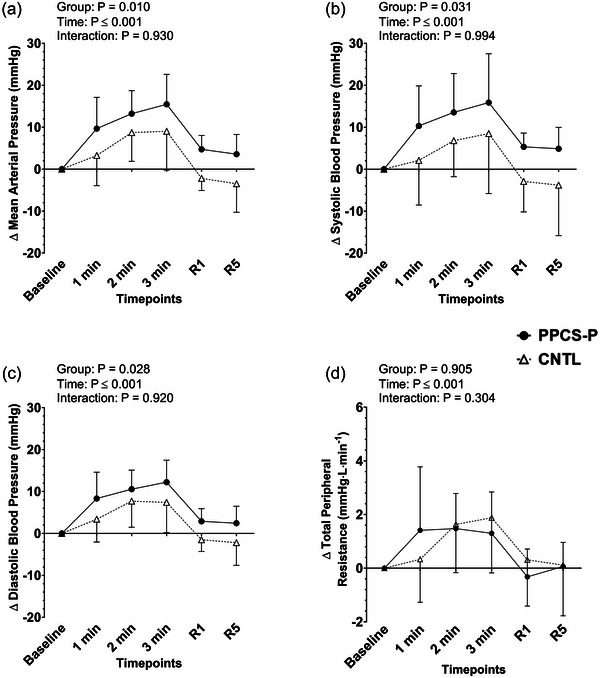
Statistical outputs from 2 (Group) × 5 (Time) linear mixed model, means and standard deviation for mean arterial pressure (a, PPCS‐P: *n* = 9, CTRL: *n* = 10), systolic blood pressure (b, PPCS‐P: *n* = 9, CTRL: *n* = 10), diastolic blood pressure (c, PPCS‐P: *n* = 9, CTRL: *n* = 10), and total peripheral resistance (d, PPCS‐P: *n* = 8, CTRL: *n* = 10). * indicates significant difference at specific timepoint.

Due to equipment issues or signal artifacts, blood pressure data was reduced to *n *= 9 for PPCS‐P and *n* = 10 for CTRL. Overall, MAP (Figure 3a), SBP (Figure 3b) and DBP (Figure 3c) all demonstrated a time effect (all *P* ≤ 0.001), but no interaction. In general, blood pressure increased during the FCPT (*P* = 0.004 for 1 min compared to 3 min for MAP) and decreased during recovery (*P* ≤ 0.001 for R5 compared to 3 min for MAP). All blood pressure variables were higher in PPCS‐P compared to CTRL (group effects all *P* ≤ 0.031). Total peripheral resistance (Figure 3d, *n* = 8 for PPCS‐P, *n* = 10 CTRL) demonstrate a time effect, with no significant differences between groups, nor interaction.

**FIGURE 4:**
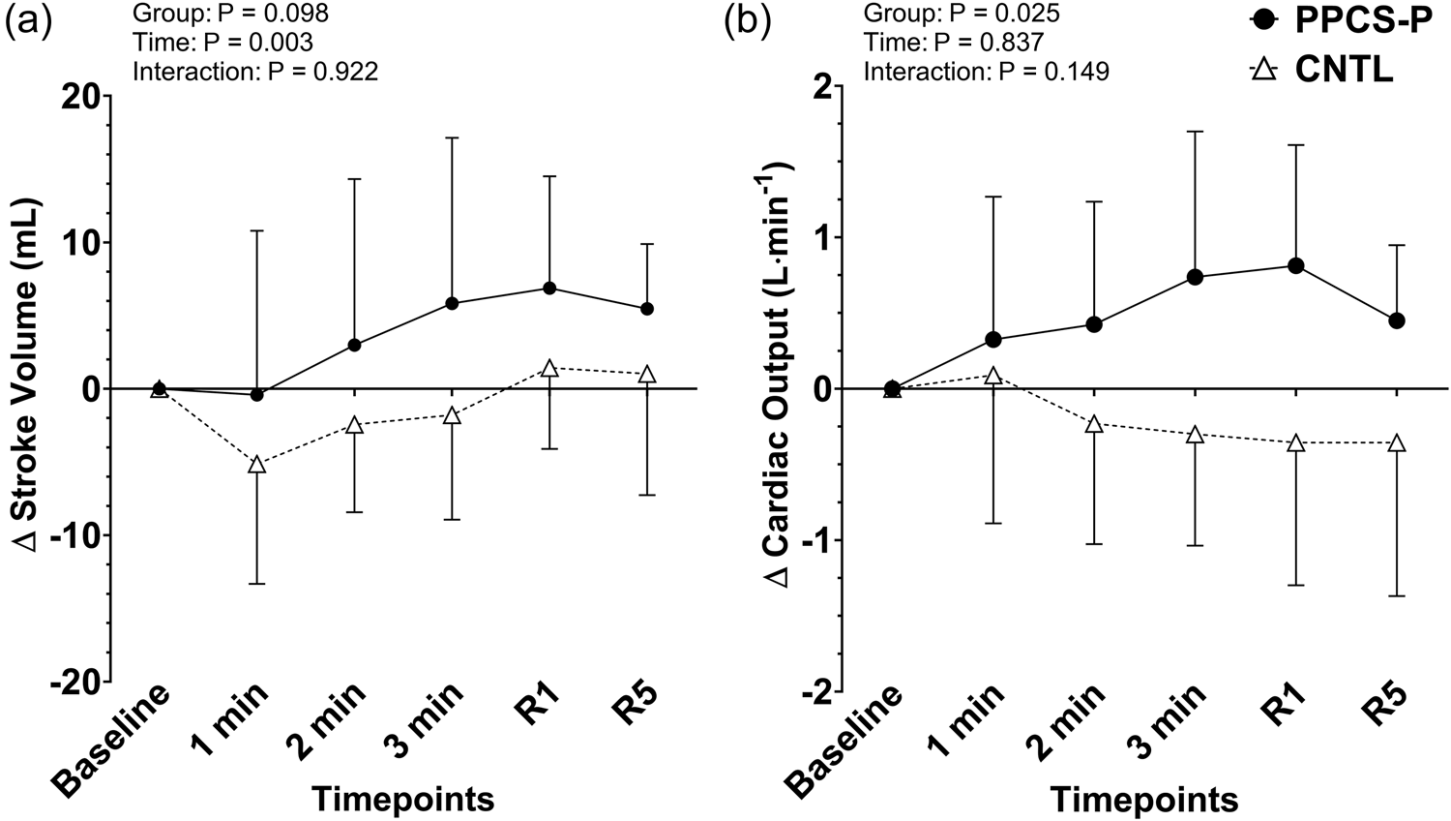
Statistical outputs from 2 (Group) × 5 (Time) linear mixed model, means and standard deviation for stroke volume (a, PPCS‐P: *n* = 8, CTRL: *n* = 10) and cardiac output (b, PPCS‐P: *n* = 8, CTRL: *n* = 10). * indicates significant difference at specific timepoint.

Due to equipment issues and signal artifacts, cardiac output and stroke volume data were reduced to *n *= 8 for PPCS‐P and *n* = 10 for CTRL. There was a time effect for stroke volume (Figure 4a), which was higher in R1 (*P* = 0.003) and R5 (*P* = 0.021) compared to 1 min only. However, there was no group or interaction effect. Cardiac output (Figure 4b) demonstrated a group effect where PPCS‐P was higher compared to CTRL, but there were no time or interaction effects.

**FIGURE 5:**
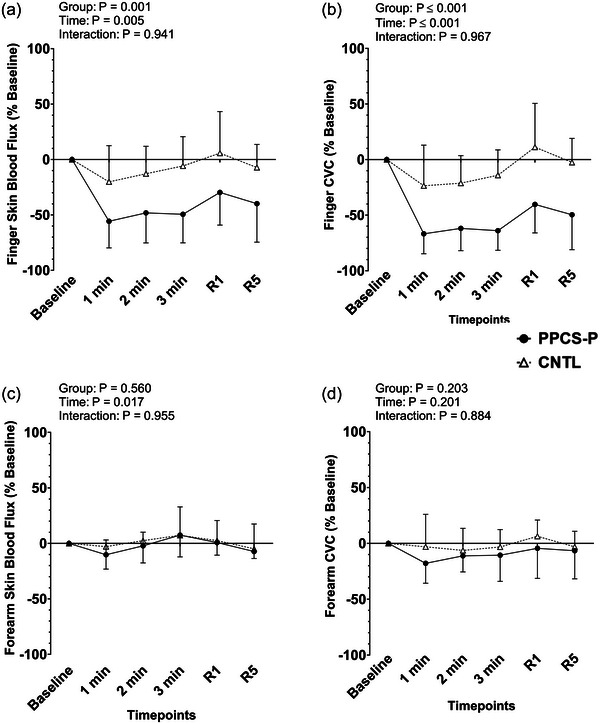
Statistical outputs from 2 (Group) × 5 (Time) linear mixed model, mean and standard deviations for percentage change in finger skin blood flux (a, PPCS‐P: *n* = 11, CTRL: *n* = 11), percentage change in finger cutaneous vascular conductance (CVC) (b, PPCS‐P: *n* = 9, CTRL: *n* = 10), percentage change in forearm skin blood flux (c, PPCS‐P: *n* = 9, CTRL: *n* = 10), and percentage change in forearm CVC (d, PPCS‐P: *n* = 7, CTRL: *n* = 10). * indicates significant difference at specific timepoint.

Finger skin blood flux (Figure 5a, *n *= 11 in PPCS‐P, *n* = 11 in CTRL) and finger CVC (Figure 5b, *n *= 9 in PPCS‐P, *n* = 10 in CTRL) both demonstrated a group and time effect, and both were lower in PPCS‐P compared to CTRL. There were no differences between groups for forearm skin blood flux (Figure 5c, *n *= 9 in PPCS‐P, *n* = 10 in CTRL) or forearm CVC (Figure 5d, *n *= 7 in PPCS‐P, *n* = 10 in CTRL). There was a time effect for forearm skin blood flux, however, when expressed as forearm CVC, there was no longer a time effect.

### 3.5 Cerebrovascular responses

**FIGURE 6:**
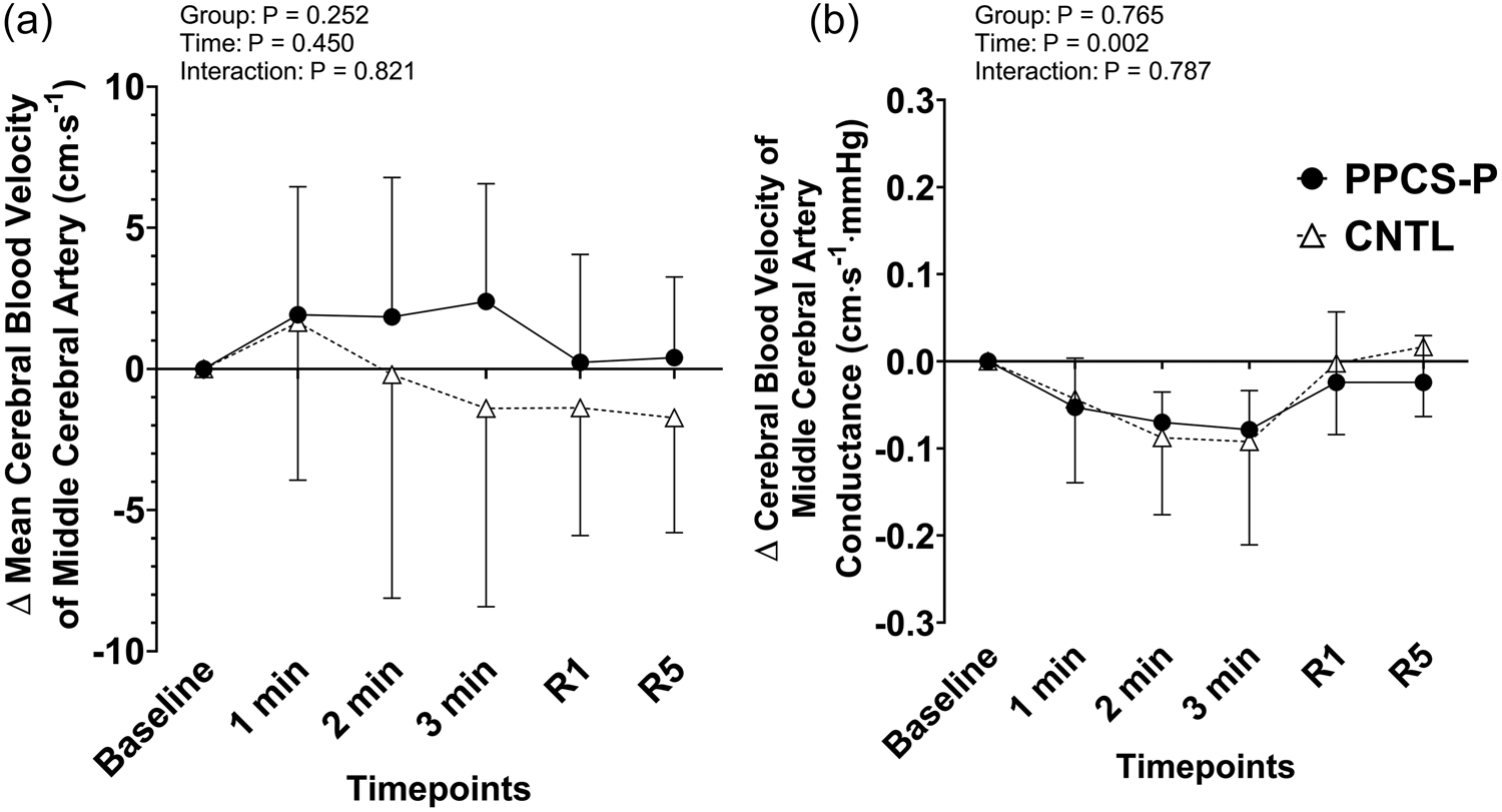
Statistical outputs from 2 (Group) × 5 (Time) linear mixed model, means and standard deviation for mean cerebral blood velocity in the middle cerebral artery (a, PPCS‐P: *n* = 9, CTRL: *n* = 10) and cerebral blood velocity in the middle cerebral artery conductance (b, PPCS‐P: *n* = 7, CTRL: *n* = 9). * indicates significant difference at specific timepoint.

There was no time, group, or interaction effect for MCA_v,mean_ (Figure 6a, *n *= 9 in PPCS‐P, *n* = 10 in CTRL). There was a time effect for MCA_v,mean‐conductance_ (Figure 6b, *n *= 7 in PPCS‐P, *n* = 9 in CTRL), which was lower at 2 min compared to R5 (*P* = 0.025), and lower at 3 min compared to R1 (*P* = 0.036) and R5 (*P* = 0.011). There were no differences between groups, nor interaction for MCA_v,mean‐conductance_.

### 3.6 Symptom responses

There was a difference (*P* = 0.016) in VAS between Baseline (3 (1.25–3.75)) and 3 min (3 (2.25–4)) of the FCPT in PPCS‐P. Six participants demonstrated no change in VAS, five participants demonstrated a 1‐point change, and one participant demonstrated a 3‐point change that subsided in recovery. There were no differences (*P* = 0.861) in post‐concussion symptom scores pre‐ (12 (8–21.5)) to post‐ (11 (5.5–24.5)) FCPT.

## 4 DISCUSSION

This study provides new insight regarding the impact of PPCS‐P on neural regulation of the human cardiovascular system: (1) the primary finding from this study is that compared with healthy controls, individuals with PPCS‐P exhibited altered cardiac and peripheral circulatory responses to trigeminal nerve stimulation using face cooling, and (2) there are no differences in cerebrovascular regulation to face cooling. Therefore, this study demonstrates that PPCS‐P alters cardiac autonomic and cardiovascular responses to face cooling when both branches of the autonomic nervous system are stimulated.

### 4.1 The impact of PPCS‐P on cardiovascular regulation

Similar to studies in adults who were recently (∼7–10 days) (Johnson et al., 2018) or remotely (>1 year) (Haider et al., 2020) concussed, we demonstrated altered cardiac autonomic regulation during FCPT in individuals with PPCS‐P. However, this response has not been seen in adolescent athletes (aged 13–18 years) within the first 10 days of injury (Haider et al., 2025). There were no differences in cardiovascular variables between PPCS‐P and CTRL at rest (Table 2), whereas altered autonomic activity became apparent using face cooling as a model of parasympathetic and sympathetic activation. Overall, nadir heart rate during face cooling was less in PPCS‐P compared to CTRL. Furthermore, heart rate decreased across the FCPT and recovery in CTRL; however, it remained unchanged throughout face cooling and recovery in PPCS‐P.

Both groups demonstrated an increase in indirect measures of sympathetic and parasympathetic activity with face cooling as indicated through an increase in blood pressure, total peripheral resistance, and cutaneous vasoconstriction (Al‐Ani et al., 1995; Brown et al., 2003; Fisher et al., 2015; Haider et al., 2020; Heath & Downey, 1990; Heindl et al., 2004; Johnson et al., 2017; Schlader et al., 2016). There was a greater increase in blood pressure throughout FCPT and recovery in PPCS‐P compared to CTRL. This is in contrast to our hypothesis and an earlier report that found that recently concussed individuals (∼7–10 days) demonstrated a blunted blood pressure response to both FCPT (Johnson et al., 2018) and a sympathetically mediated hand cold pressure test (Johnson et al., 2020). Recently, it has been demonstrated that the MAP response during face cooling was not different between adolescents who sustained a sports‐related concussion within 10 days of injury, and the response was similar for those who were confirmed to develop persisting post‐concussion symptoms (Haider et al., 2025). Furthermore, in healthy adults (Schlader et al., 2016) and individuals who are recently concussed (∼7–10 days) (Johnson et al., 2018), cardiac output and stroke volume are largely maintained throughout face cooling and not different from healthy controls. However, in our study, cardiac output was higher, and stroke volume was not different in PPCS‐P compared to CTRL. Therefore, in our study, the greater blood pressure responses to facial cooling appears to be attributed due to the higher cardiac output (due to higher heart rates) as oppose to differences in total peripheral resistance. Future studies would benefit by utilizing the sympathetically mediated hand cold pressure test to isolate the sympathetic branch of the autonomic nervous system to better understand differences in cardiac autonomic function and blood pressure responses in PPCS‐P.

Face cooling causes a reflex vasoconstriction response in peripheral skin (i.e. fingers) for ∼120 s that decreases finger blood flux and returns to baseline (along with MAP) when cold stress is removed in healthy adults (Brown et al., 2003; Gagnon et al., 2013, Mugele et al., 2023). This response is more pronounced in glabrous skin (i.e. fingerpad), which is primarily regulated by the adrenergic vasoconstrictor system, whereas non‐glabrous skin (i.e. forearm) is regulated through the adrenergic vasoconstrictor system and multiple pathways (i.e. co‐transmitters, inhibition of nitric oxide vasodilation, α_2c_‐adrenergic receptor expression) (Greaney et al., 2015; Hodges et al., 2019, 2006). In our study, there was a sustained decrease in finger skin blood flux and finger CVC in PPCS‐P during the FCPT and recovery, where these variables did not return to baseline following 5 min of recovery in PPCS‐P. There were minimal changes in forearm skin blood flux or forearm CVC, nor were there differences between groups. Therefore, in PPCS‐P, the sustained decrease in finger skin blood flux may suggest an exaggerated efferent cutaneous sympathetic response to FCPT (Hilz et al., 1999). However, future studies are needed using microneurography to directly measure sympathetic outflow to determine these mechanistic pathways. Furthermore, we did not examine vasoconstriction in other vascular beds (e.g. renal, splanchnic, muscle) which can influence MAP. Despite these findings, one research consideration is that hands were not warmed before, during or after the FCPT. As blood pressure was determined from finger photoplethysmography, the decreased finger skin blood flux may have contributed to this response. Future research should incorporate both finger photoplethysmography and automatic brachial artery occlusion during the FCPT to confirm blood pressure responses.

We used heart rate variability measures as an indirect indicator of vagal‐mediated cardiac parasympathetic activity during trigeminal nerve stimulation using face cooling. Similar to individuals who are acutely concussed (Johnson et al., 2018), have a remote history of concussion (Haider et al., 2020) or who have familial dysautonomia (Hilz et al., 1999), individuals with PPCS‐P demonstrated a blunted heart rate variability response during face cooling compared to healthy controls. Specifically, there were minimal changes in RMSSD and pNN50 during face cooling and recovery, whereas these values increased in CTRL. Specifically, these time domain analyses of heart rate variability suggest that the smaller heart rate reductions among individuals with PPCS‐P were attributed to lesser cardiac parasympathetic outflow and/or greater cardiac sympathetic outflow during the FCPT (Hilz et al., 1999). Although the CTRL group increased heart rate variability during FCPT (i.e. RMSSD and pNN50), the RMSSD response was lower in magnitude than previous studies (Haider et al., 2020; Johnson et al., 2018; Schlader et al., 2016). With face cooling, the cooling temperature, the size of the exposed region, and the amount of cutaneous thermal receptors stimulated determines the magnitude of the FCPT response (Heath & Downey, 1990). In our study, the cold stimulus was only applied to the bilateral cheeks and nose (due to the transcranial doppler head piece covering the forehead), as opposed to cheeks, nose, eyes and forehead (Haider et al., 2020; Johnson et al., 2018; Schlader et al., 2016), which likely contributed to the lower magnitude of the RMSSD response. However, despite these methodological differences, there were differences between groups in heart rate and heart rate variability indicating that there is altered cardiac autonomic activity to face cooling in PPCS‐P. Collectively, our results demonstrated alterations in cardiac autonomic nervous system activation and peripheral vascular responses to face cooling with individuals with PPCS‐P.

### 4.2 Face cooling on the regulation of cerebral blood velocity in middle cerebral artery

We found no differences in MCA_v,mean_ or MCA_v,mean‐conductance_, indicating no differences in cerebral blood velocity responses during face cooling in PPCS‐P compared to CTRL. Individuals with PPCS‐P are demonstrated to have a reduced sensitivity to arterial carbon dioxide leading to disproportionate elevations in MCA_v,mean_ during exercise compared to healthy controls, which is restored with sub‐symptom aerobic exercise training (Clausen et al., 2016). Furthermore, adolescent athletes who are concussed (38–61 days post‐injury), demonstrate impaired dynamic cerebral autoregulation during orthostatic changes from sitting to standing, which also improved in some patients following 6 weeks of standard medical care (Moir et al., 2018). As well, the pressure‐buffering capacity of the middle cerebral artery during repeated squats is reduced ∼2 weeks following a sports‐related concussion despite clinical recovery and then resolves ∼30 days post‐injury (Wright et al., 2018). In healthy controls, cerebral autoregulation appears to remain intact during face cooling, as there are slight increases in MCA_v,mean_ in the first 60 s of cold exposure (Brown et al., 2003). We aimed to determine if there were differences in cerebral blood velocity in the middle cerebral artery responses to face cooling in PPCS‐P as it challenges the autonomic nervous system, cardiovascular system and involves dynamic changes in peripheral and cerebral vascular resistance (Brown et al., 2003). However, despite the higher MAP compared to CTRL and increased total peripheral resistance, MCA_v,mean‐conductance_ decreased to help maintain MCA_v,mean_, which suggests a preserved cerebral autoregulation during face cooling. Participants were allowed to freely breathe and were instructed to not hold their breath to avoid the Valsalva manoeuvre during the FCPT to avoid confounding effects of changes in arterial carbon dioxide. The FCPT has been previously demonstrated to have a minimal change in end‐tidal carbon dioxide values (Brown et al., 2003). Combined, the FCPT may not be a sufficient stressor on cerebrovascular regulation due to minimal changes in arterial carbon dioxide and/or pressure, and thus the null differences between groups. The classic diving response occurs with a combination of both cold‐face stimulation (water) with breath holding (i.e. apnoeas) where there is increased cerebral blood flow due to increases in arterial carbon dioxide (Baranova et al., 2016). It would be interesting to determine if there is impaired cerebral autoregulation in the diving reflex with breath holding and face cooling in PPCS‐P because it would stimulate both branches of the autonomic nervous system, as well as lead to changes in arterial carbon dioxide and MCA_v,mean_. This would provide an integrated and mechanistic understanding of how changes in autonomic nervous system function and arterial carbon dioxide influence cerebral blood flow regulation in PPCS‐P.

### 4.3 Future directions

Future research should investigate the mechanisms of sub‐symptom aerobic exercise on cardiac autonomic function in PPCS‐P using tests that stress the autonomic nervous system (Mercier et al., 2022) such as the FCPT, hand cold pressor test (to isolate the sympathetic branch of the autonomic nervous system) (Johnson et al., 2020), or orthostatic stress (Balestrini et al., 2021; Moir et al., 2018; Wright et al., 2018). Recently, Mercier et al. (2024) performed a 12‐week sub‐symptom aerobic exercise training programme (5×/week, 70–80% sub‐symptom threshold, 30 min, cardio‐based exercise, revised using BCTT every 3 weeks) in individuals with PPCS‐P (*n* = 50, diagnosed mild traumatic brain injury ≥3 months to ≤5 years since injury) and found no differences in resting heart rate or heart rate variability pre‐ to post‐intervention. Potentially there was a null effect on cardiac autonomic activity, as there was not sufficient autonomic strain on the participants (Mercier et al., 2022). We hypothesize if sub‐symptom aerobic exercise can restore autonomic balance, there should be an improvement in the FCPT response as indicated by an improved nadir heart rate response. Recently, Haider et al. (2025) demonstrated no change in FCPT responses at clinical recovery, which included individuals who recovered from a sports‐related concussion and those who had persisting post‐concussion symptoms (physiological, vestibular‐occular, cervogenic, cognitive/mood). However, comparisons between different sub‐types of persisting post‐concussion symptoms were not made. This may also point to physiological recovery lagging behind clinical recovery (Haider et al., 2025; Patricios et al., 2023; Wright et al., 2018). Therefore, future studies may benefit from performing autonomic tests at clinical recovery and continuing to test post‐clinical recovery (e.g. 1 month) as part of their research design.

### 4.4 Experimental considerations

Due to the nature of this population, there will be individual variability with PPCS‐P for level of recovery/impairment. Each participant was diagnosed with a sports‐related concussion (previous 12 months) and persisting post‐concussion symptoms by a physician and referred by Brock University's Sports Medicine Clinic to progress to aerobic exercise training. However, some of the individuals may have previously been or were receiving concurrent cervical treatment (i.e. exercise rehab, manual treatment) and/or vision therapy exercises. We did not control for the type and duration of concurrent care in this study. Therefore, some of the participants may have additionally had cervogenic and/or neurovestibular/ocular persisting post‐concussion symptoms, as opposed to purely PPCS‐P. We did not perform a FCPT on individuals with persisting post‐concussion symptoms without exercise intolerance; however, this would have allowed us to compare against individuals with different subtypes or who may have recovered. There is a debate between studies on the use of ultra‐short (1 min) heart rate variability sampling times for time domain measures at rest compared to 5 min recordings. Some studies show no differences and excellent (>0.90) intraclass correlations for RMSSD (Esco & Flatt, 2014; Holmes et al., 2020; Munoz et al., 2015; Wu et al., 2020), while others show good intraclass correlations for RMSSD and pNN50 for 1–2 min samples compared to 5 min (Burma et al., 2021). Differences between studies may be due to resting time prior to measures, or differences in body position (Holmes et al., 2020). To investigate this issue, we compared the impact of sample duration on the calculation of heart rate and heart rate variability. We found no differences (all *P* > 0.05) between 1 min and 5 min sampling durations for heart rate (0.0 ± 2.0 beats·min^−1^ difference), RMSSD (−2.3 ± 10.9 ms difference), and pNN50 (−0.0 ± 9.0% difference). Based on these *post hoc* analyses, we suspect that there was a low likelihood that sampling duration contributed to the between group differences in this study. A *post hoc* power calculation (G*Power 3.1.9.7) was performed on the primary outcome variable, nadir heart rate during FCPT, which demonstrated an effect size of *d* = 1.05, with a power of β = 0.69. In order to achieve an α = 0.05 and β = 0.80, we would require *n* = 16 in each group. The measures of stroke volume are indirect (Van Lieshout et al., 2003), and we are unable to determine the absolute differences between groups, and therefore these data better reflect difference in stroke volume change with FCPT between groups. We did not have a direct measurement of sympathetic outflow (i.e. muscle or skin sympathetic nerve activity). Lastly, we did not have any neuroimaging or assessments of cardiac function, and cannot rule out brainstem abnormalities and damage to cardiovascular control centres which have been demonstrated following a concussion (Dobson et al., 2017; Levine et al., 2008) influencing responses to face cooling.

### 4.5 Conclusion

Overall, we demonstrated alterations in cardiac and peripheral autonomic nervous system activity during a model of parasympathetic and sympathetic activation using face cooling in individuals with PPCS‐P compared to apparently healthy controls. Specifically, there were group differences where there was a blunted nadir heart rate response, blunted heart rate variability (RMSSD, pNN50), higher blood pressure, higher cardiac output and sustained lower finger skin blood flux responses in PPCS‐P compared to CTRL. There were no differences in MCA_v,mean_ or MCA_v,mean‐conductance_ between groups. Overall, these findings demonstrate that there are alterations in systemic physiological regulation with PPCS‐P that extend outside of the brain, including the altered neural control of the cardiovascular system.

## AUTHOR CONTRIBUTIONS

Phillip J. Wallace conceived the experiment. Phillip J. Wallace, Steve Lidstone, Brandon J. McKinlay, Stephen A. Klassen, and Stephen S. Cheung contributed to the design of the study. Phillip J. Wallace, Steve Lidstone, Brandon J. McKinlay, Josh G. Nowlan, Johnathan Ljubanovich, and Stephen S. Cheung piloted and performed the experiments. Phillip J. Wallace performed the statistical analysis. All authors interpreted the results of the study. Phillip J. Wallace drafted the manuscript. All authors edited and revised the final version of the manuscript. All authors have read and approved the final version of this manuscript and agree to be accountable for all aspects of the work in ensuring that questions related to the accuracy or integrity of any part of the work are appropriately investigated and resolved. All persons designated as authors qualify for authorship, and all those who qualify for authorship are listed.

## CONFLICT OF INTEREST

The authors declare that the research was conducted in the absence of any commercial or financial relationships that could be construed as a potential conflict of interest.

## Data Availability

The data to support the findings of this study are available from the corresponding author upon reasonable request.
